# Environmental Enrichment and Estrogen Upregulate Beta-Hydroxybutyrate Underlying Functional Improvement

**DOI:** 10.3389/fnmol.2022.869799

**Published:** 2022-05-03

**Authors:** Soonil Pyo, Joohee Kim, Jihye Hwang, Jeong Hyun Heo, Kyungri Kim, Sung-Rae Cho

**Affiliations:** ^1^Department and Research Institute of Rehabilitation Medicine, Yonsei University College of Medicine, Seoul, South Korea; ^2^Brain Korea 21 Plus Project for Medical Sciences, Yonsei University College of Medicine, Seoul, South Korea; ^3^Graduate Program of Biomedical Engineering, Yonsei University College of Medicine, Seoul, South Korea; ^4^Department of Physiology, Yonsei University College of Medicine, Seoul, South Korea; ^5^Rehabilitation Institute of Neuromuscular Disease, Yonsei University College of Medicine, Seoul, South Korea

**Keywords:** environmental enrichment (EE), sex, beta-hydroxybutyrate (β-HB), estrogen, female, functional improvement, brain derived neurotrophic factor (BDNF), neuroplasticity

## Abstract

Environmental enrichment (EE) is a promising therapeutic strategy in improving metabolic and neuronal responses, especially due to its non-invasive nature. However, the exact mechanism underlying the sex-differential effects remains unclear. The aim of the current study was to investigate the effects of EE on metabolism, body composition, and behavioral phenotype based on sex. Long-term exposure to EE for 8 weeks induced metabolic changes and fat reduction. In response to the change in metabolism, the level of βHB were influenced by sex and EE possibly in accordance to the phases of estrogen cycle. The expression of β-hydroxybutyrate (βHB)-related genes and proteins such as monocarboxylate transporters, histone deacetylases (HDAC), and brain-derived neurotrophic factor (BDNF) were significantly regulated. In cerebral cortex and hippocampus, EE resulted in a significant increase in the level of βHB and a significant reduction in HDAC, consequently enhancing BDNF expression. Moreover, EE exerted significant effects on motor and cognitive behaviors, indicating a significant functional improvement in female mice under the condition that asserts the influence of estrogen cycle. Using an ovariectomized mice model, the effects of EE and estrogen treatment proved the hypothesis that EE upregulates β-hydroxybutyrate and BDNF underlying functional improvement in female mice. The above findings demonstrate that long-term exposure to EE can possibly alter metabolism by increasing the level of βHB, regulate the expression of βHB-related proteins, and improve behavioral function as reflected by motor and cognitive presentation following the changes in estrogen level. This finding may lead to a marked improvement in metabolism and neuroplasticity by EE and estrogen level.

## Introduction

Sex differences in metabolic responses to exercise have been presented in many previous studies (Davis et al., 2000; Kang et al., 2006; Vislocky et al., 2008; Hagobian et al., 2009; Isacco and Miles-Chan, 2018). During a prolonged exercise, females oxidize more lipids and fewer carbohydrate than males oxidize, resulting in greater lipolysis (Tarnopolsky, 2008; Henderson, 2014; Maunder et al., 2018; Allman et al., 2019). These differences are partially due to the higher circulating concentrations of estrogen in females (Gavin et al., 2013; Brockman and Yardley, 2018). Estrogen controls lipolysis in various tissues, and its beneficial effect on whole-body fat reduction has been reported (Pedersen et al., 2004; D'eon et al., 2005; Gavin et al., 2013). At a rate proportional to fat oxidation, ketogenesis occurs mainly in the mitochondria of liver cells (Lopes-Cardozo et al., 1975). It is caused by nutrient-deficient conditions such as caloric restriction (Lin et al., 2015), fasting (Balasse and Fery, 1989; Higashino-Matsui et al., 2012), and prolonged exercise (Phinney, 2004; Hargreaves and Spriet, 2020), which breaks down free fatty acids to produce ketone bodies such as acetoacetate and β-hydroxybutyrate (βHB; Dhillon and Gupta, 2020).

Environmental enrichment (EE) is a method of raising rodents in a huge cage, with novel objects and running wheels, and allowing numerous social interactions (Nithianantharajah and Hannan, 2006). Previous studies have demonstrated that exposure to EE can elicit energetic stress with diverse cellular responses to maintain energy homeostasis (Goodpaster and Sparks, 2017; De Souza et al., 2019; Queen et al., 2020) and modulate systemic metabolism, increase the rate of metabolic processes, and modulate levels of various metabolites (Fery and Balasse, 1983; Mika et al., 2019; Thyfault and Bergouignan, 2020). EE exerts beneficial effects on behaviors and emotions in various rodent models (Matsumori et al., 2006; Nithianantharajah and Hannan, 2006; Madronal et al., 2010) via improvement of brain metabolism (Takimoto and Hamada, 2014; Matsui et al., 2017; Puchalska and Crawford, 2017). Moreover, these beneficial effects of EE are highly influenced by biological sex (Davis et al., 2000; Lin et al., 2011; Kiss et al., 2013; Chamizo et al., 2016).

Under nutritional stress after starvation or exercise, the synthesis and utilization of ketone bodies in hepatic mitochondria through free fatty acid metabolism can produce necessary nutrients to extrahepatic tissues such as the brain (Evans et al., 2017; Puchalska and Crawford, 2017). Endogenous βHB can easily cross the blood-brain barrier (Hasselbalch et al., 1995). However, the permeability of βHB in neuronal cells is dependent on the expression of monocarboxylate transporters (MCTs; Pierre and Pellerin, 2005; Vijay and Morris, 2014), and the permeated βHB is further catalyzed by 3-hydroxybutyrate dehydrogenase as the first stage of βHB oxidation (Evans et al., 2017). Among the endogenous ketone bodies, βHB can act as an epigenetic regulator through the inhibition of histone deacetylases (HDACs) in the brain, which in turn increases the acetylation of histones occupying loci related to brain-derived neurotrophic factor (BDNF). Previous studies have presented similar results that βHB can act as an epigenetic regulator in various cell types (Dabek et al., 2020; Zhang et al., 2020; Ruppert et al., 2021). More specifically, βHB can modulate the expression of BDNF with histone modifications on several locations (Xie et al., 2016; Hu et al., 2020; Mierziak et al., 2021). Mounting evidence suggests that changes in the level of βHB following EE and estrogen may regulate BDNF level in an epigenetic manner.

Although several studies have examined the metabolic and neuronal responses to EE, less attention has been paid to the mechanism underlying the effects of EE on metabolism and its brain function under the influence of estrogen cycle. Therefore, the purpose of this study is to determine whether EE exerts effects on metabolism under the influence of estrogen.

## Materials and Methods

### Ethics Statement and Experimental Animals

All procedures were reviewed and approved by the Association for Assessment and Accreditation of Laboratory Animal Care and the Institutional Animal Care and Use Committee of the Yonsei University Health System (permit number: 2018-0110, 2019-0336, and 2020-0054). All procedures were in accordance with the guidelines of the National Institutes of Health's Guide for the Care and Use of Laboratory Animals. These regulations, notifications, and guidelines originated and were modified from the Animal Protection Law (2008), Laboratory Animal Act (2008), and Eighth Edition of the Guide for the Care and Use of Laboratory Animals (NRC 2011). Mice were provided food and water *ad libitum* under a 12-h light/dark cycle, according to animal protection regulations. They were sacrificed at 8 weeks after the special housing conditions under anesthesia induced by intraperitoneal injection of ketamine (100 mg/kg) and xylazine (10 mg/kg) anesthesia by intraperitoneal injection. All efforts were made to minimize animal suffering.

### Experimental Procedures and Cage Condition

At 6 weeks of age, a total of 104 (52 male and 52 female) ICR/CD-1 mice were randomly housed in either standard conditions (SC, *n* = 26 per sex) or an enriched environment (EE, *n* = 26 per sex). Additionally, a total of 70 mice underwent ovariectomy [OVX group, *n* = 24; OVX + Estradiol (E2) group, *n* = 15; OVX + EE group, *n* = 14; and OVX + E2/EE, *n* = 17]. The subcutaneous injection of E2 treatment (5 μg E2/corn oil) was administered every 3–4 days. The treatments (E2 and/or EE) for each group lasted until 14 weeks of age, as previously described (Seo et al., 2018). All mice were fed a normal chow diet, and vaginal cytology was conducted for all female mice immediately before sacrifice, as previously described (Byers et al., 2012).

### Dual Energy X-Ray Absorptiometry

Dual Energy X-Ray Absorptiometry (DXA) is a method to assess *in vivo* body composition in humans and animals, and can differentiate between fat and non-fat tissues. DXA was performed at 14 weeks of age to determine the whole-body composition of each group of mice. Each mouse was anesthetized with a mixture of xylazine and ketamine, transferred, and correctly positioned in the DXA chamber, and bone mineral content, fat, and lean body mass were measured.

### Blood Biochemical Testing

All mice went through fasting process for 5–6 h before blood collection. Blood was drawn into BD Microtainer^®^ blood collection tubes (USA). Blood samples were centrifuged for 10 min at 300 g, and serum supernatants were collected and transferred to other tubes. Total protein, total cholesterol, high-density lipoprotein, triglyceride, and lactate dehydrogenase levels were measured using DRI-CHEM 4000i (Fuji).

### Indirect Calorimetry

To investigate the effect of EE on metabolism and physiology under the influence of estrogen, a total of 12 mice (*n* = 3 per group) for the normal model and a total of 22 OVX mice (OVX group, *n* = 6, OVX + E2 group, *n* = 6; OVX + EE group, *n* = 5, OVX + E2/EE, *n* = 5) were housed in metabolic automatic cages (Phenomaster, TSE systems) at 14 weeks of age. After 2 days of acclimation, measurements of energy expenditure, respiratory exchange ratio, and core temperature were taken at 9-min intervals for a total of 5 days.

### βHB Assay

βHB levels were measured in the blood serum and brain tissue extracts using the βHB assay kit from Sigma-Aldrich (MAK041-1KT) following the manufacturer's instructions. The values were obtained at 1:40 dilution and expressed as pmol/well, unless noted otherwise; each well contained a total volume of 200 μL.

### Quantitative Real-Time PCR

Total RNA was prepared in the interested brain tissue lysates using TRIzol reagent (Invitrogen Life Technologies, Carlsbad, CA, USA) according to the manufacturer's instructions. A nanodrop spectrophotometer (Thermo Fisher Scientific, Waltham, MA, USA) was used to confirm the quality and quantity of extracted RNA. Differentially expressed genes of interest from cerebral cortex and hippocampus were selected to validate by Quantitative Real-Time PCR (qRT-PCR). ReverTra Ace^®^ qPCR RT Master Mix with gDNA Remover (Toyobo, Osaka, Japan) was used to synthesize cDNA with total RNA. Then, 2 μl of cDNA in a total volume of 20 μl was used in the following reaction. The qRT-PCR was performed in triplicate on a Light Cycler 480 (Roche Applied Science, Mannheim, Germany) using the Light Cycler 480 SYBR Green master mix (Roche), with thermocycler conditions as follows: amplifications were performed starting with a 300 s template preincubation step at 95°C, followed by 45 cycles at 95°C for 10 s, 60°C for 10 s, and 72°C for 10 s. The melting curve analysis began at 95°C for 5 s, followed by 1 min at 60°C. The specificity of the produced amplification product was confirmed by the examination of a melting curve analysis and showed a distinct single sharp peak with the expected Tm for all samples. A distinct single peak indicates that a single DNA sequence was amplified during qRT-PCR. The detail sequence of the primers is listed in Supplementary Table 1. Primers were designed using the NCBI primer blast with the parameters set to a product of 150–200 bp within the region surrounding the identified translocation. The expression of each gene of interest was obtained using the 2^−Δ*ΔCT*^ method. The expression level of each gene of interest was obtained using the 2^−Δ*ΔCT*^ method. Target-gene expression was normalized relative to the expression of GAPDH and represented as fold change relative to the male control group.

### Western Blot

Brain lysates were isolated from cerebral cortex and hippocampus. To assess the protein expression of MCT1, MCT2, MCT4, HDAC1, HDAC2, HDAC3, BDNF in cerebral cortex and hippocampus, total protein was extracted from all mice and dissolved in sample buffer (60 mM Tris–HCl, pH 6.8, 14.4 mM b-mercaptoethanol, 25% glycerol, 2% SDS, and 0.1% bromophenol blue; Invitrogen), incubated for 10 min at 70°C, and separated on a 10% SDS reducing polyacrylamide gel (Invitrogen). Twenty microgram of Protein samples were separated with SDS-polyacrylamide gel electrophoresis (PAGE) on a 4–12% gradient Bis-Tris gel and Tris-Acetate gel (Invitrogen, Carlsbad, CA, USA). The separated proteins were further transferred onto a 0.45 μm Invitrolon^TM^ polyvinylidene difluoride (PVDF) filter paper sandwich using a XCell II^TM^ Blot Module (Invitrogen, Life Technologies, Carlsbad, CA, USA). The membranes were blocked for 1 h in Tris-buffered saline (TBS) (10 mM Tris-HCl, pH 7.5, 150 mM NaCl) plus 0.05% Tween 20 (TBST) containing 5% non-fat dry milk (Bio-Rad, Hercules, CA, USA) at room temperature, washed three times with TBST, and incubated at 4°C overnight with the following primary antibodies; anti-MCT1 (1:500, Abcam), anti-MCT2 (1:200, Santa Cruz), anti-MCT4 (1:200, Santa Cruz), anti-HDAC1 (1:500, Santa Cruz), anti-HDAC2 (1:200, Santa Cruz), anti-HDAC3 (1:1,000, Santa Cruz), anti-BDNF (1:1,000, Abcam), and anti-β-Actin (1:5,000, Abcam). After washing the blots three times with TBST, the blots were incubated for 1 h with horseradish peroxidase-conjugated secondary antibodies (1:5,000; Santa Cruz, CA, USA) at room temperature. The proteins were further washed three times with TBST and visualized with an enhanced chemiluminescence (ECL) detection system (Amersham Pharmacia Biotech, Little Chalfont, UK). Using ImageQuant™ LAS 4000 software (GE Healthcare Life Science, Chicago, IL, USA), western blot results were saved into TIFF image files, and then the images and the density of the band were analyzed and expressed as the ratio relative to the control band density using Multi-Gauge (Fuji Photo Film, version 3.0, Tokyo, Japan). To normalize the values of all samples to account for band intensity, the average band intensity for each mouse group was first calculated. The samples were normalized to the group average of controls, and target protein expressions were normalized relative to the internal expression of β-Actin. The value of the male control group was set to 1 and was divided by the value of each individual mouse.

### Immunohistochemistry

The brain tissues were frozen in Surgipath FSC 22 clear frozen section compound (Leica Microsystems) using dry ice and isopentane. The harvested brain tissues were cryosectioned into 16-μm-thick sections along the coronal plane, and immunohistochemical staining was performed as previously described. Eight weeks after EE, to confirm the endogenous expression of MAP2 (1:400, Abcam), GFAP (1:400, Neuromics), MCT2 (1:400, Santa Cruz), and MCT4 (1:400, Santa Cruz), the brain sections of the cerebral cortex and hippocampus were immunostained. The sections were incubated with Alexa Fluor^®^ 488 goat anti-rabbit (1:400, Invitrogen) and Alexa Fluor^®^ 594 goat anti-mouse (1:400, Invitrogen) secondary antibodies, and then covered with Vectashield^®^ mounting medium with 4C, 6-diamidino-2-phenylindole (Vector, Burlingame, CA, USA). The stained sections were analyzed using an LSM 700 confocal microscope (Zeiss, Gottingen, Germany). The z-stack confocal analysis was used to measure the colocalization of MAP2 with MCT2 and GFAP with MCT4 and to create Maximum Intensity Projection (MIP) and Ortho (2.5D) images for further colocalization clarification.

### Behavioral Assessments

#### Rotarod Test

A rotarod test was used to assess motor coordination and locomotor function. All animals received a pretreatment performance evaluation at 6 weeks of age. For this assessment, mice were placed on a rotarod treadmill [Ugo Basile, Gemonio (VA), Italy], and the latency to fall, which is the length of time that the animals remained on the rolling rod, was measured. Rotarod tests were then performed at a 2-week interval until 14 weeks of age after the commencement of the treatment at an accelerating speed (4–80 rpm) and a constant speed (32, 64 rpm). The latency period was measured twice for each test, and individual tests were terminated at a maximum latency of 300 s.

#### Y-Maze Test

Y-maze test is to evaluate cognition and short-term spatial memory (Wahl et al., 1992). This test was carried out in an enclosed “Y” shaped maze (Jeung Do B&P). Normal mice tend to visit the arms of the maze one after the other. This behavior is called spontaneous alternation and used to assess short-term spatial memory in a new environment (Wahl et al., 1992). The number of each arm entries, spontaneous alternation, and percent alternation were recorded for 8 min. The percent alternation was calculated as follows: [Number of spontaneous alternation/(Number of total arm entries – 2)] × 100. At the end of each trial the maze was cleaned of urine and feces with 70% ethanol.

#### Hanging Wire Test

Hanging wire test is designed to assess both limb strength and balance (Manwani et al., 2011). Mice were tested for their ability to hang from a thick metallic wire, which was secured to two vertical stands. To avoid any vibration, the wire was tightly attached to the plastic frame. The wire was secured 35 cm above the bedding cage to prevent falling injury. Each mouse was put on three trials. Latency to fall from the wire was recorded, and the maximum latency period was 300 s.

#### Open Field Test

Open field test is generally used to evaluate locomotor activity and anxiety behaviors in a novel environment (Kraeuter et al., 2019). Activity monitoring was conducted on a premise of square area measuring 30 × 30.5 × 31 cm^3^. The dimension of the premise was divided into 16 sectors. The 4 inner sectors were marked as the center, while the 12 outer sectors were defined as the periphery. Total spent time in the periphery was recorded as an index of anxiety. Mice were placed individually into the periphery of the area and could explore freely for 25 min while being monitored with a video camera. The data were analyzed using the video tracking system Smart Vision 2.5.21 (Panlab, Barcelona, Spain).

### Statistical Analysis

Statistical analyses were performed using the Statistical Package for Social Sciences software (version 25.0; IBM Corporation, Armonk, NY, USA). Levene's test was applied for the homogeneity of variance of the data. A two-way analysis of variance (ANOVA) test was used to examine the primary effects (Sex or Period or Housing) and interaction effects (Sex × Housing or Period × Housing), followed by Tukey's *post-hoc* test for comparisons within sex or period and between housing conditions under significant interaction. A two-way repeated measure ANOVA test was used to examine the primary and interaction effects within and between groups (5 × 4 factorial design) for the rotarod test. *Post-hoc* analysis was used to find where the significant differences were, and was identified at *p* < 0.01 using a Bonferroni adjustment as a multiple pairwise comparison. For the comparison of the OVX groups, a one-way ANOVA followed by Tukey's multiple comparison test was used. The data are expressed as the mean ± standard error of the mean (SEM), and all graphical artworks were produced using GraphPad Prism version 9.

## Results

### EE Induces Metabolic Changes and Fat Reduction

**Figure 1:**
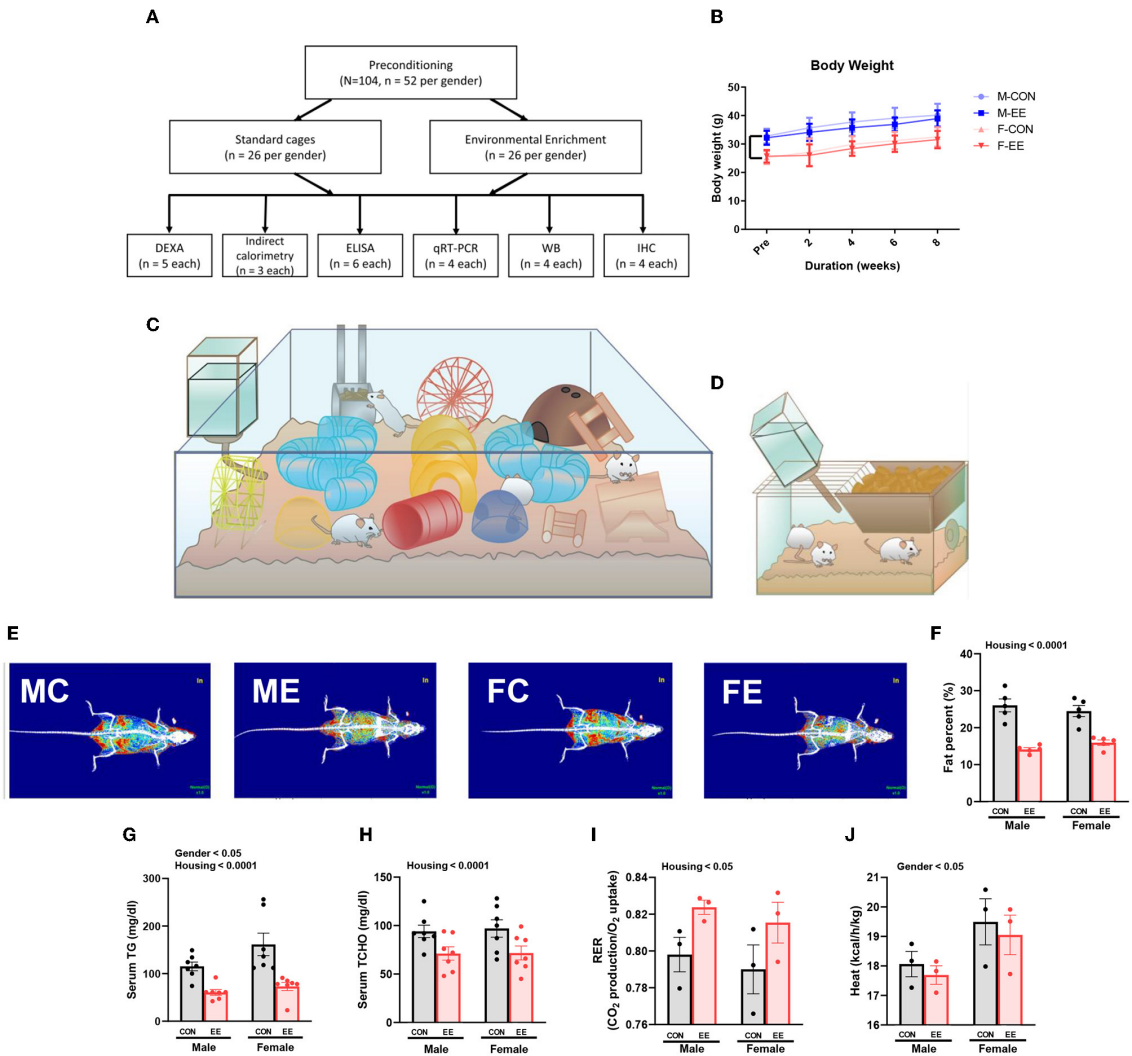
Differential metabolic change by sex sensitivity and exposure to EE in normal models. **(A)** The experimental scheme for normal models. **(B)** Body weight change at 2-week interval (*n* = 26 per group). The representative figures of **(C)** EE cage and **(D)** control cage. **(E)** The representative DXA image of each group. **(F)** Percentage fat in male and female mice exposed to control (CON) or enriched (EE) cages (*n* = 5 per group). After exposure to EE or control cage, blood biochemical analysis (*n* = 7 per group) and indirect calorimetry (*n* = 3 per group) were conducted. **(G)** Serum triglyceride. **(H)** Serum total cholesterol. **(I)** Respiratory exchange ratio. **(J)** Heat. Two-way ANOVA with Tukey multiple comparison test. Significant sex effect, significant housing effect, and the significant interaction between sex and housing were noted with *p*-value. Data are means ± SEM.

For 8 weeks from 6 weeks of age, normal mice were exposed to either control cages or EE cages (Supplementary Figure 1A); the experimental scheme for normal models is shown in Figures 1A,C,D, and body weight change is noted in Figure 1B. Representative DXA images from each group are shown in Figure 1E. In DXA images, red dots indicate fat tissues, blue dots indicate non-fat tissues, and white dots indicate bone tissues. Significant housing effect was observed, indicating that fat reduction occurred after exposure to EE regardless of sex (Figure 1F). In blood biochemical analysis, significant gender effect and housing effect were noted in serum triglyceride (TG), indicating that EE mice have lower TG level than control mice regardless of sex, while female mice showed a higher TG level than male mice regardless of housing condition (Figure 1G). A significant housing effect in serum total cholesterol (TCHO) was also observed, indicating that exposure to EE can decrease TCHO level regardless of sex (Figure 1H). Metabolic cage analysis showed a significant housing effect in the respiratory exchange ratio, indicating that EE mice have higher RER ratio than control mice regardless of sex (Figure 1I) and significant sex effect on heat, indicating that female mice have higher heat than male mice regardless of housing condition (Figure 1J). Collectively, these data imply that female mice have different body composition compared to male mice, and long-term exposure to EE can induce fat reduction and change body composition, with resultant metabolic changes in both sexes of mice.

### The Level of βHB Were Influenced by Sex and EE in Normal Mice

**Figure 2:**
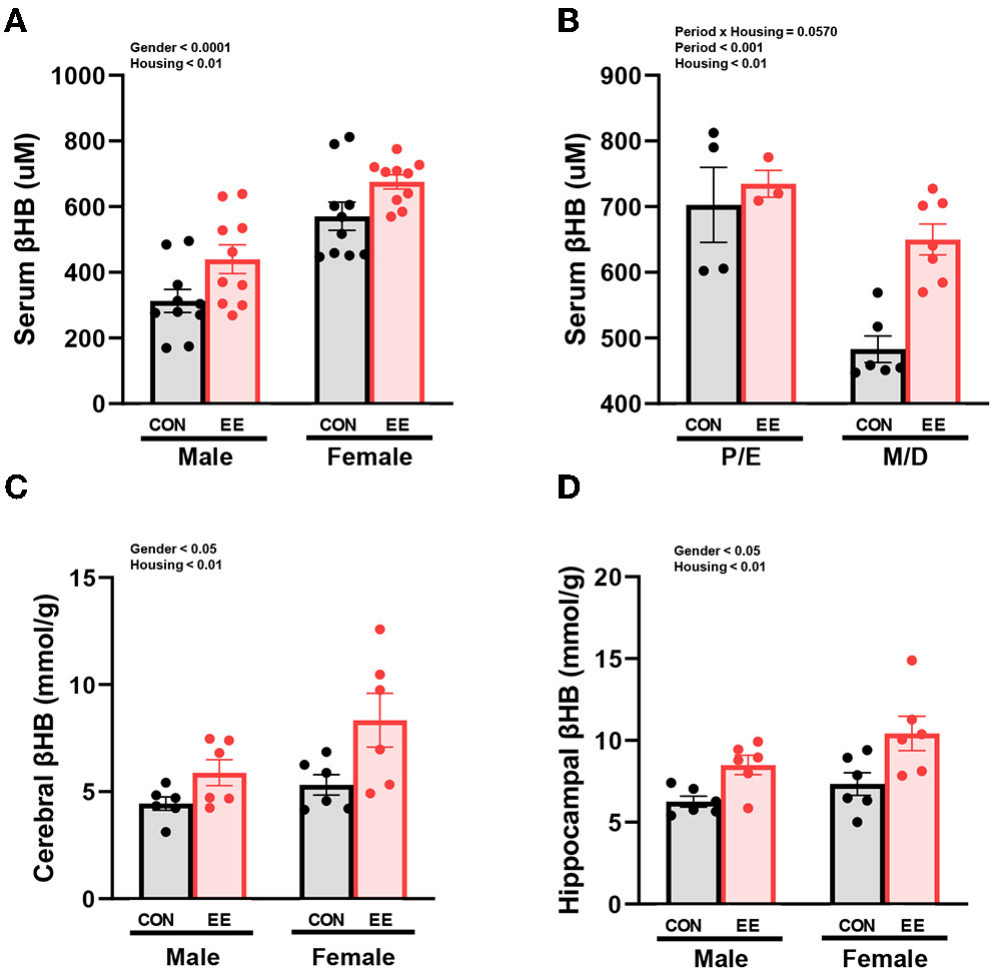
Differential βHB level by sex and exposure to EE in normal models. βHB enzyme-linked immunosorbent assay (ELISA) was conducted in serum (*n* = 10 per group), cerebral cortex (*n* = 6 per group), and hippocampus (*n* = 6 per group). **(A)** Serum βHB levels. **(B)** Further female subgroup analysis based on estrus cycle in serum βHB. **(C)** Cerebral cortex βHB and **(D)** Hippocampus βHB. Two-way ANOVA with Tukey multiple comparison test. Significant sex effect, significant housing effect, and the significant interaction between sex and housing were noted with *p*-value. Data are means ± SEM.

βHB enzyme-linked immunosorbent assay (ELISA) analysis indicated significant housing and sex effects on serum βHB levels (Figure 2A), indicating that female mice have higher βHB level than male mice regardless of housing, and EE mice have higher βHB level than control mice regardless of sex. Further analysis based on the estrus cycle indicated a trend on the interaction between housing and period (*P* = 0.0570). Significant housing effect and period effect on serum βHB levels in female mice were observed, indicating that serum βHB levels in proestrus and estrus (P/E) stage were higher than in metestrus and diestrus (M/D) stage regardless of housing, and EE mice have higher serum βHB levels than control mice regardless of period (Figure 2B). The stage of the estrus cycle was determined through vaginal cytology (Supplementary Figure 1B). Moreover, significant housing and sex effects were also indicated on βHB levels in the cerebral cortex and hippocampus, respectively (Figures 2C,D). This indicates that female mice have higher βHB levels than male mice regardless of housing, and EE mice have higher βHB levels than control mice regardless of sex. These data demonstrate that the baseline level of βHB is different between sexes, and EE can induce metabolic changes and alter body composition. The changes in female mice are due to the influence of the estrus cycle in response to the increased rate of lipolysis.

### The Expression of βHB-Related Genes and Proteins Were Influenced by Sex and EE

**Figure 3:**
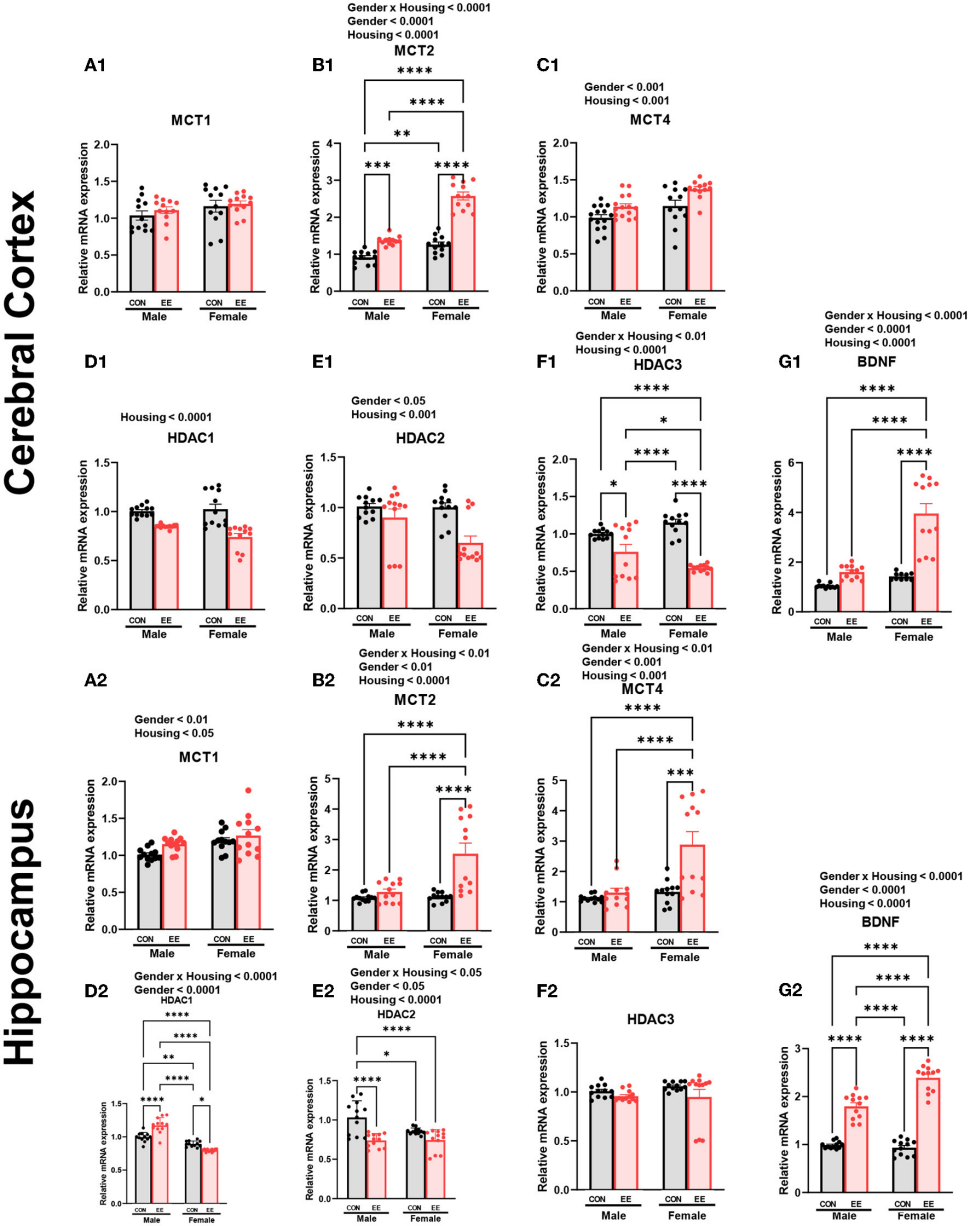
Effects of EE on the expression of βHB-related genes under the influence of estrogen by qRT-PCR. **(A1–G1)** Cerebral cortex and **(A2–G2)** hippocampus mRNA levels for βHB-related genes measured using qRT-PCR (*n* = 4 per group). All samples were run in triplicate. Two-way ANOVA with Tukey multiple comparison test. Significant sex effect, significant housing effect, and the significant interaction between sex and housing were noted with *p*-value. Data are means ± SEM. *p < 0.05, **p < 0.01, ***p < 0.001, and ****p < 0.0001.

**Figure 4:**
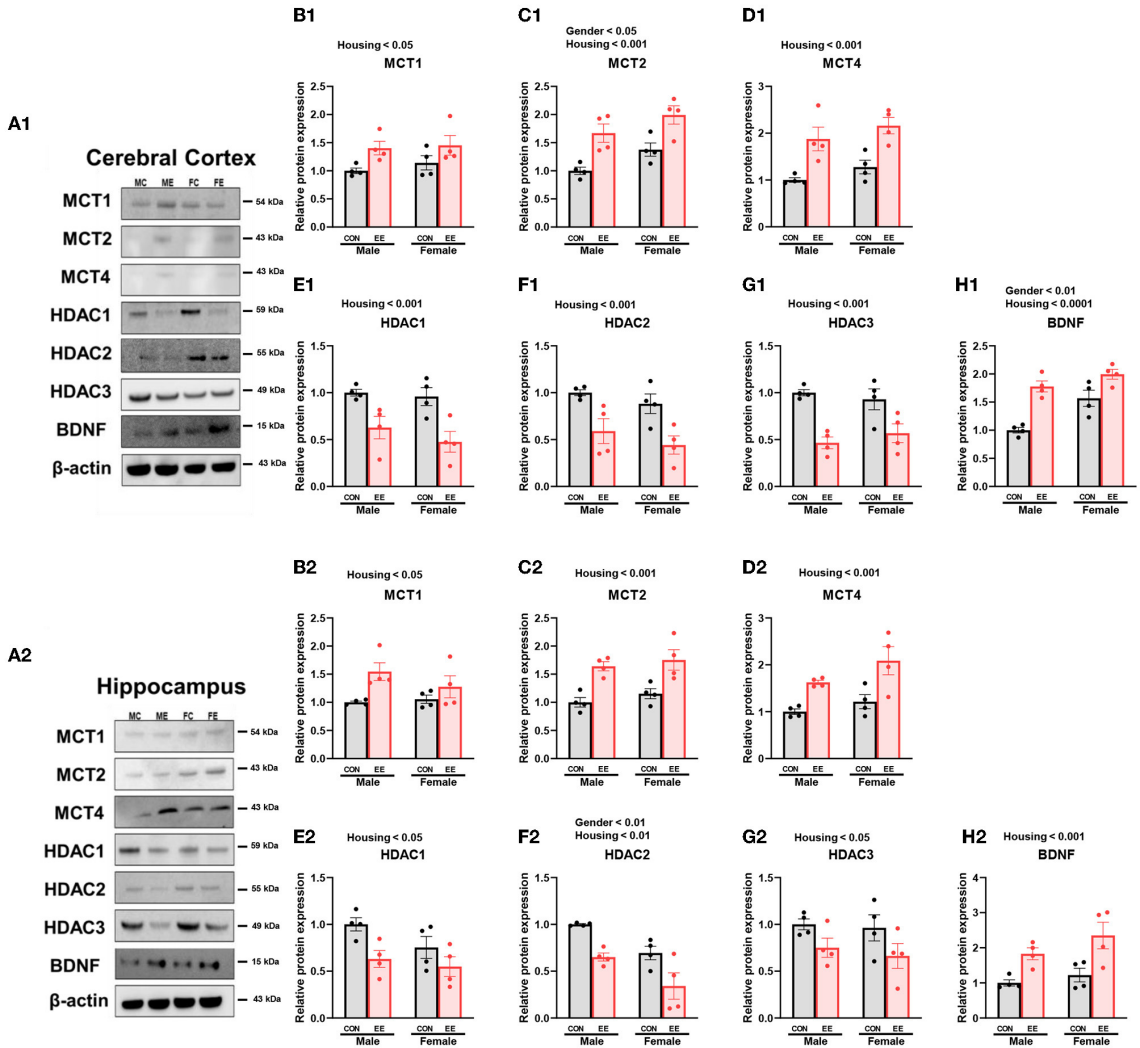
Effects of EE on the expression of βHB-related proteins under the influence of estrogen by western blot. **(A1–H1)** Cerebral cortex and **(A2–H2)** hippocampus protein levels for βHB-related proteins measured using western blot (*n* = 4 per group). Two-way ANOVA with Tukey multiple comparison test. Significant sex effect, significant housing effect, and the significant interaction between sex and housing were noted with *p*-value. Data represented are means ± SEM.

To examine the expression of βHB-related genes, qRT-PCR was conducted in the cerebral cortex (Figures 3A1–G1) and hippocampus (Figures 3A2–G2). There was a significant interaction between housing and gender in MCT2 [*F*_(1, 44)_ = 36.18, *P* = 0.0001], HDAC3 [*F*_(1, 44)_ = 10.96, *P* = 0.0019], and BDNF [*F*_(1, 44)_ = 20.85, *P* = 0.0001] in cerebral cortex. Moreover, there was a significant interaction between housing and gender in MCT2 [*F*_(1, 44)_ = 11.38, *P* = 0.0016], MCT4 [*F*_(1, 44)_ = 8.822, *P* = 0.0048], HDAC1 [*F*_(1, 44)_ = 42.80, *P* = 0.0001], HDAC2 [*F*_(1, 44)_ = 5.344, *P* = 0.0255], and BDNF [*F*_(1, 44)_ = 29.65, *P* = 0.0001] in hippocampus. Significant housing effects and sex effects are noted in the figures. Western blotting was conducted to examine the expression of βHB-related proteins in cerebral cortex (Figures 4A1–H1) and hippocampus (Figures 4A2–H2), and representative images are shown in Figures 4A1,A2, respectively. Significant housing or sex effect was noted in βHB-related proteins. Collectively, these data indicate that the expression of βHB-related genes and proteins was significantly regulated by EE and sex. In cerebral cortex and hippocampus, EE led to a significant increase in the level of βHB and a significant reduction in the level of HDAC, which consequently enhanced BDNF expression under the influence of sex.

### EE Induces Higher βHB Uptake of the Brain by Both Astrocytes and Neurons

**Figure 5:**
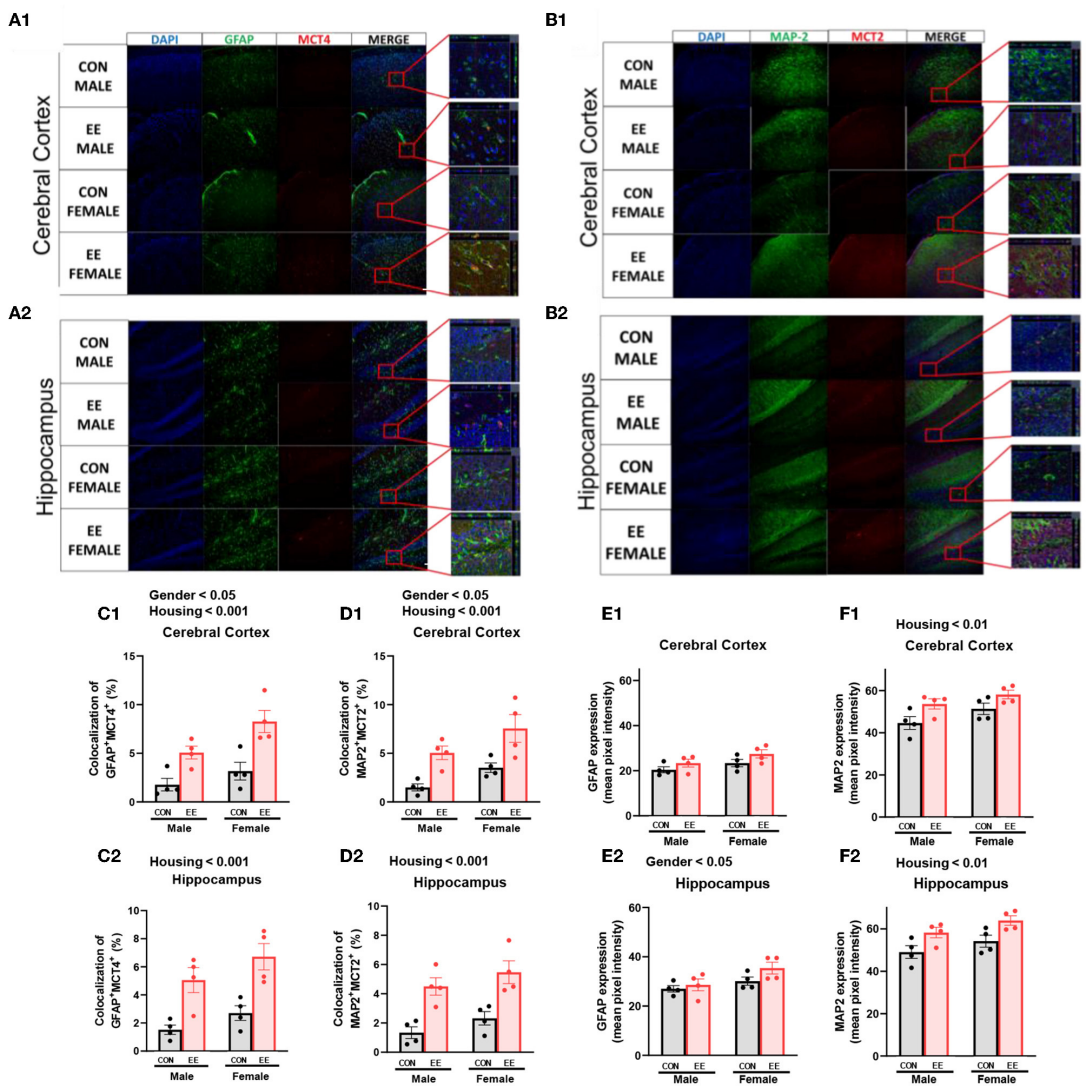
Effects of EE on the uptake of βHB in cerebral cortex and hippocampus under the influence of estrogen. Representative IHC images of **(A1**,**A2)** GFAP^+^MCT4^+^ in cerebral cortex and hippocampus and **(B1**,**B2)** MAP2^+^MCT2^+^ in the cerebral cortex and hippocampus (*n* = 4 per group). **(C1–F1)** Quantification of GFAP^+^MCT4^+^ and MAP2^+^MCT2^+^ in the cerebral cortex and hippocampus. **(C2–F2)** Raw intensity of GFAP and MAP2 in cerebral cortex and hippocampus. Two-way ANOVA with Tukey multiple comparison test. Significant sex effect, significant housing effect, and the significant interaction between sex and housing were noted with *p*-value. Data are means ± SEM.

To examine the uptake of βHB in cerebral cortex and hippocampus, histological assessments with GFAP, MCT4, MAP-2, and MCT2 were conducted. The representative images of GFAP and MCT4 in the cerebral cortex and hippocampus are shown in Figures 5A1,A2, respectively. The representative images of MAP-2 and MCT2 in the cerebral cortex and hippocampus are shown in Figures 5B1,B2, respectively. Significant sex and housing effect were noted both in the colocalization of GFAP and MCT4 in cerebral cortex (Figure 5C1) and the colocalization of MAP2 and MCT2 in hippocampus (Figure 5D1), indicating that female mice have the higher colocalization than male mice regardless of housing, and EE mice have the higher colocalization than control mice regardless of sex. Significant housing effect was noted in the colocalization of GFAP and MCT4 in cerebral cortex (Figure 5C2) and in the colocalization of MAP2 and MCT2 in hippocampus (Figure 5D2), implying that EE mice have higher colocalization than control mice regardless of sex. Raw intensity of GFAP and MAP2 for cerebral cortex and hippocampus are shown in Figures 5E1,F1,E2,F2. Significant housing effect was observed in raw MAP2 expression, demonstrating that EE mice showed a higher expression of MAP2 than control mice regardless of sex in cerebral cortex and hippocampus. Significant gender effect was observed in raw GFAP expression, indicating that female mice showed higher expression of GFAP than male mice regardless of housing in hippocampus. These combined data indicate that long-term exposure to EE can induce the higher βHB uptake as indicated by the higher colocalization rate in cerebral cortex and hippocampus by astrocytes and neurons.

### EE Improves the Motor and Cognitive Functions in Normal Mice

**Figure 6:**
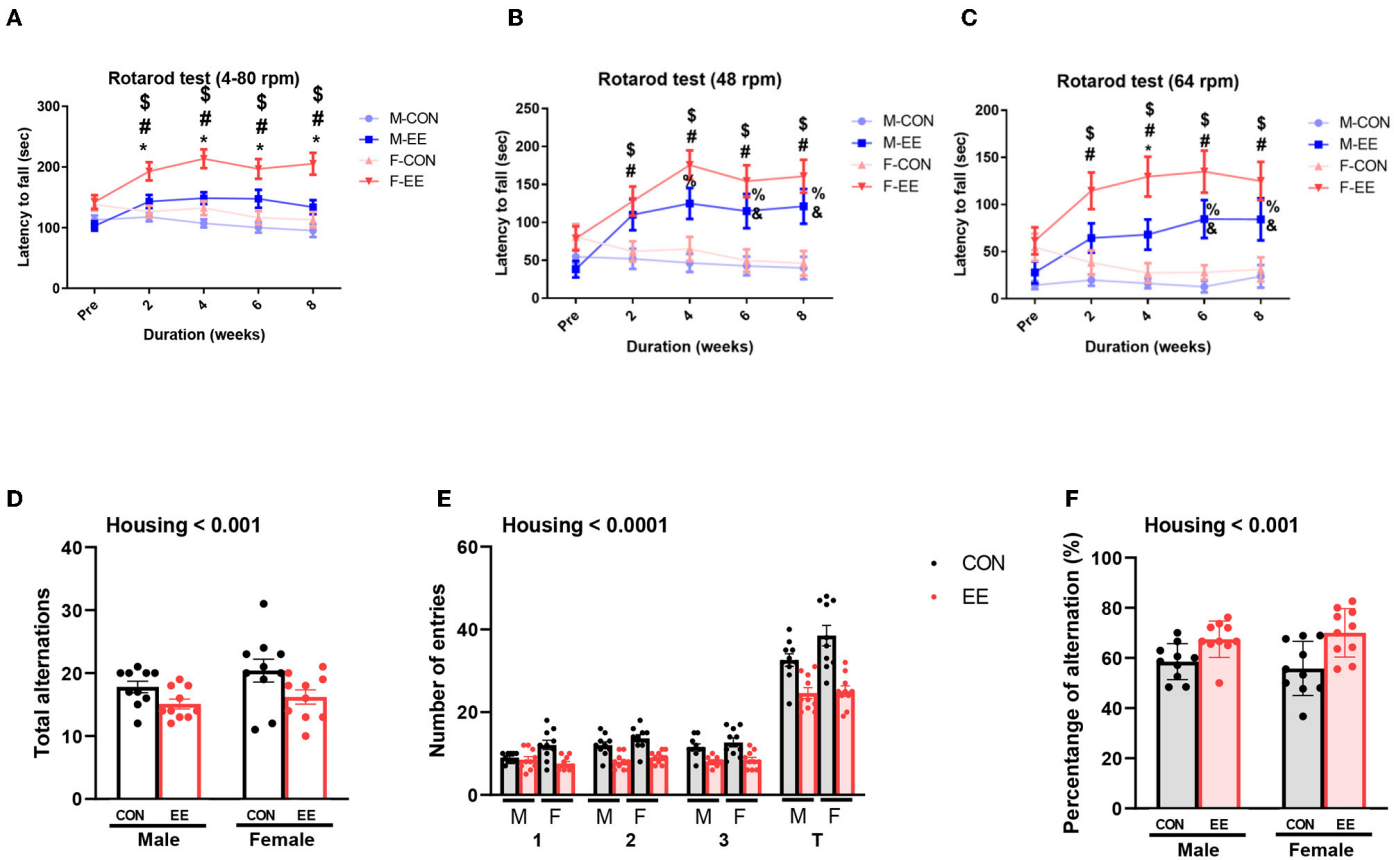
Effects of EE on functional improvement in normal models. Behavioral test assessments conducted for the normal groups. **(A)** Rotarod accelerating from 4 to 80 rpm. **(B)** Rotarod at constant 48 rpm. **(C)** Rotarod at constant 64 rpm (*n* = 26 per group). *F-EE vs. M-EE, ^#^F-EE vs. F-CON, ^$^F-EE vs. M-CON, ^%^M-EE vs. M-CON, and ^&^M-EE vs. F-CON. **(D)** Number of alternative behaviors, **(E)** number of entries, and **(F)** percent alternation of Y-maze test (*n* = 10 per group). Two-way ANOVA with Tukey multiple comparison test. Significant sex effect, significant housing effect, and the significant interaction between sex and housing were noted with *p*-value. Data represented are means ± SEM. *p < 0.01, ^#^*p* < 0.01, ^*$*^*p* < 0.01, ^%^*p* < 0.01, and ^&^*p* < 0.01.

To examine motor and cognitive function of normal mice, rotarod test and Y-maze test were conducted. In rotarod test, female EE mice significantly outperformed the other groups of mice at accelerating speed 4–80 rpm (Figure 6A), constant 48 rpm (Figure 6B), and constant 64 rpm (Figure 6C). Specifically, rotarod performance was significantly improved in EE mice from 2 to 8 weeks after EE treatment. In Y-maze test, significant housing effect was observed in the number of alterative behaviors (Figure 6D) and the number of entries (Figure 6E), demonstrating that the raw number of alternation and total entries were significantly decreased in EE mice compared to control mice regardless of gender. Significant housing effect was observed in the percentage of alternation, indicating that EE mice have higher percentage of alternation than control mice regardless of gender (Figure 6F). These data indicated that exposure to EE improves cognitive function. These combined data indicate that long-term exposure to EE improves motor and cognitive function.

### EE and Estrogen Treatment Can Induce Lipolysis and Metabolic Changes in OVX Mice

**Figure 7:**
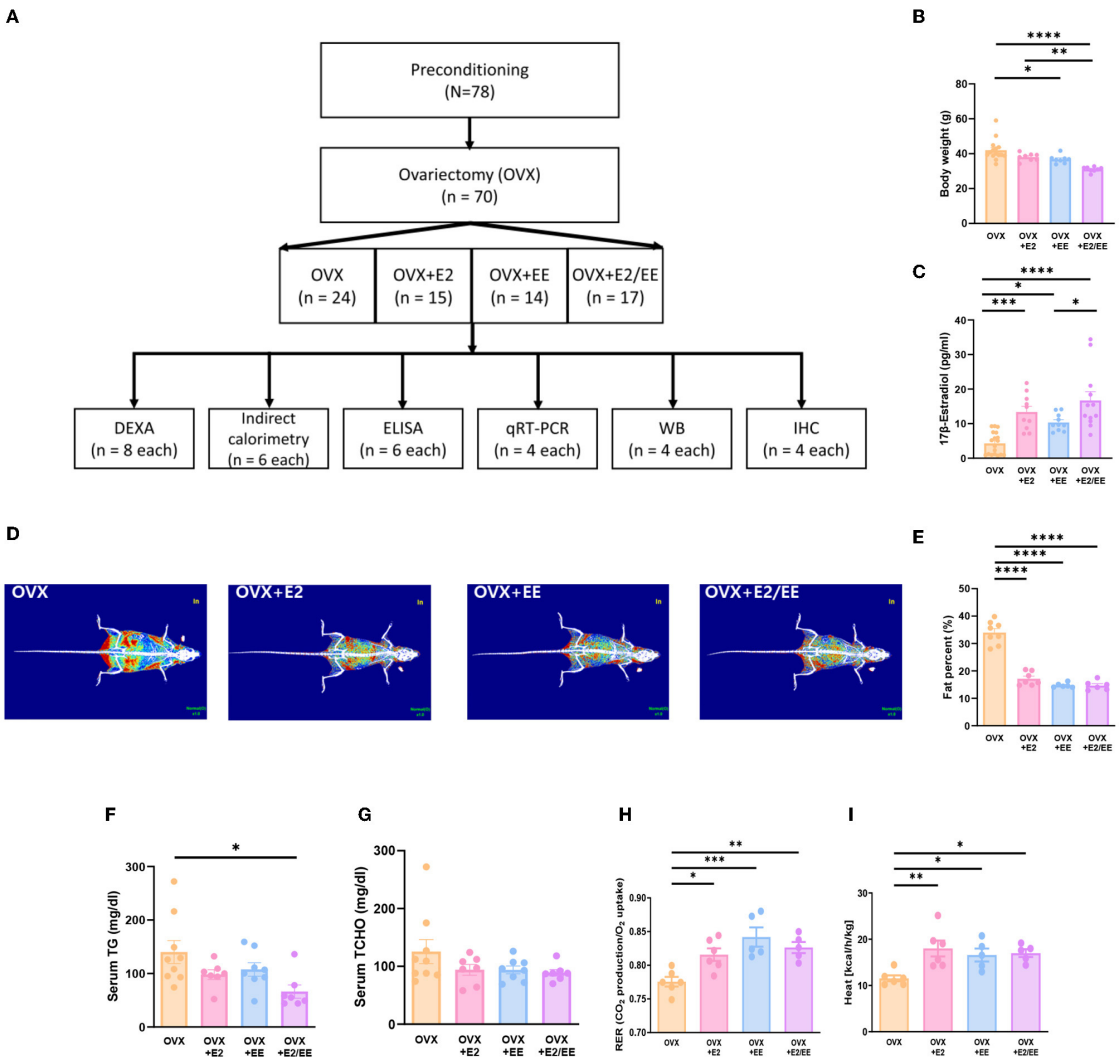
Differential metabolic change following estrogen treatment and exposure to EE in OVX models. **(A)** Experimental scheme for OVX models. **(B)** Body weight of OVX groups after the treatments (E2 and/or EE) (*n* = 10–18 per group). **(C)** Measurement of serum 17β-estradiol (*n* = 10–18 per group). **(D)** Representative DXA image from each group. **(E)** Percentage body fat (*n* = 6–8 per group). **(F)** Serum triglyceride (*n* = 7–9 per group). (**G**) Serum total cholesterol (*n* = 7–9 per group). **(H)** Respiratory exchange ratio (*n* = 5–6 per group). **(I)** Heat (*n* = 5–6 per group). One-way ANOVA with Tukey multiple comparison test. Data represented are means ± SEM. *p < 0.05, **p < 0.01, ***p < 0.001, and ****p < 0.0001.

To produce an estrogen-deficient model, OVX was conducted at 6 weeks of age (Supplementary Figure 1C), and the experimental scheme for OVX models is shown in Figure 7A. Body weight measurement for OVX group, OVX + E2 group, OVX + EE group, and OVX + E2/EE group is noted in Figure 7B, and the body weight was significantly decreased by exposure to EE and the synergistic effect of EE and E2. The level of 17β-estradiol was significantly increased by estrogen treatment and exposure to EE (Figure 7C). The representative DEXA images from each group are shown in Figure 7D, and significant fat reduction following estrogen treatment and exposure to EE was observed (Figure 7E). Moreover, in blood biochemical analysis, the synergistic effect of estrogen and exposure to EE was observed in serum TG (Figure 7F), decreasing the level of TG compared to OVX group but not in serum TCHO (Figure 7G). In indirect calorimetry, exposure to EE and estrogen treatment significantly increase RER (Figure 7H) and heat (Figure 7I) in OVX mice, indicating that both treatments can change body composition and induce metabolic changes. Collectively, exposure to EE and estrogen treatment can synergistically increase lipolysis, alter body composition, and metabolic changes in OVX mice.

### EE and Estrogen Treatment Can Reduce Abnormal Lipid Accumulation in Liver and Various Brain Regions in OVX Mice

**Figure 8:**
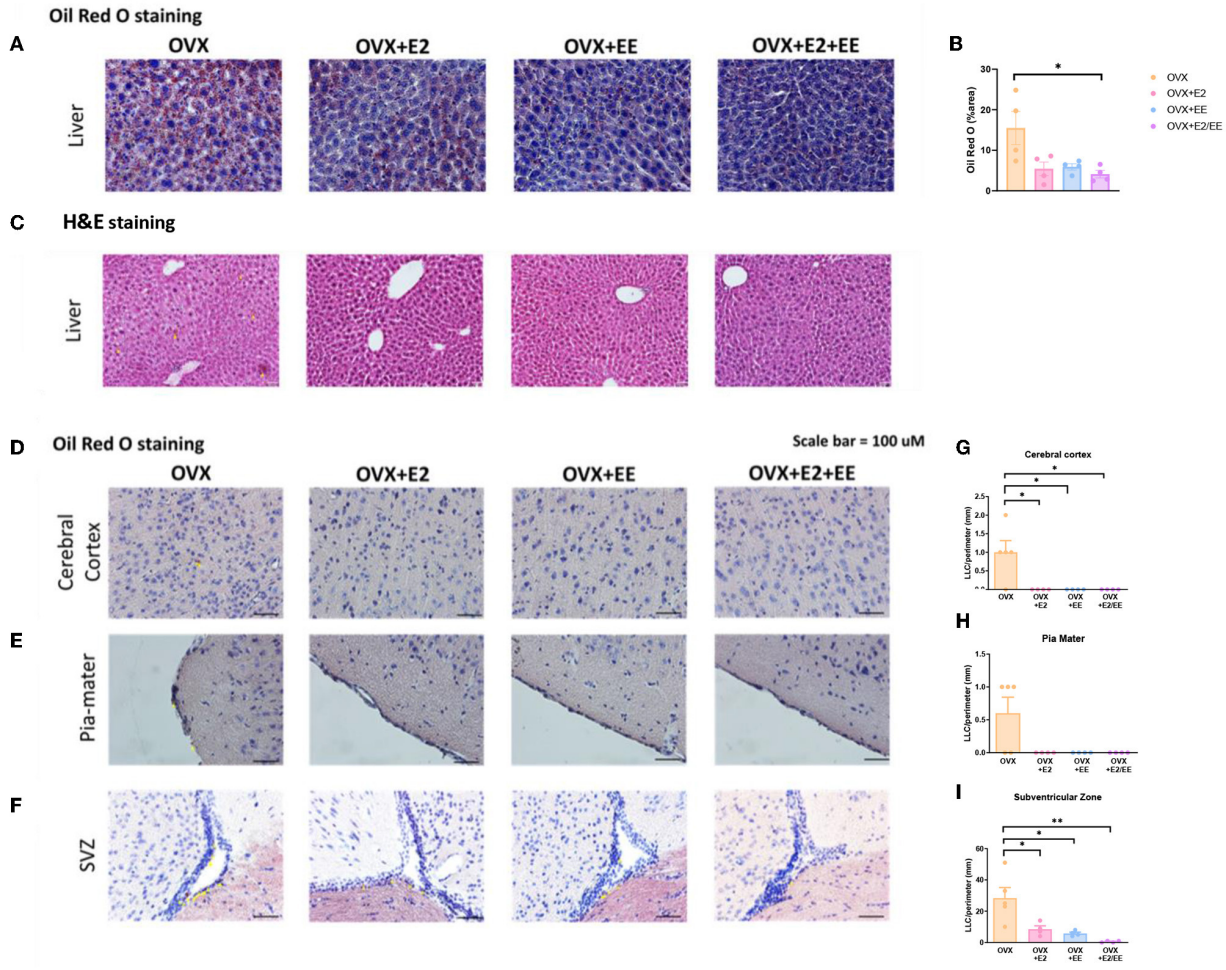
Abnormal lipid accumulation was alleviated by exposure to EE and estrogen treatment in OVX mice. **(A)** The representative images of ORO-stained liver of the OVX groups, and **(B)** quantification thereof. **(C)** The representative images of H&E-stained liver of the OVX groups. The representative images of ORO-stained **(D)** cerebral cortex, **(E)** pia-mater, and **(F)** SVZ, and **(G–I)** quantification thereof, respectively (*n* = 4 per group). One-way ANOVA with Tukey multiple comparison test. Data represented are means ± SEM. *p < 0.05 and **p < 0.01. White bars = 50 μm.

To examine lipid accumulation in the liver and various brain regions of OVX mice, Oil Red O (ORO) staining, and H&E staining were conducted. The representative images of ORO-stained livers for each group are shown in Figure 8A, and the abnormal lipid accumulation was significantly decreased by the synergistic effect of EE and estrogen treatment (*P* = 0.0194, Figure 8B). Moreover, hepatic steatosis was observed in the OVX group and was diminished by exposure to EE and/or estrogen treatment (Figure 8C). The representative images of ORO-stained cerebral cortex, pia-mater, and subventricular zone (SVZ) are shown in Figures 8D–F, respectively. The quantification of ORO-stained cerebral cortex, pia-mater, and SVZ are shown in Figures 8G–I. Exposure to EE and estrogen treatment significantly reduced the abnormal accumulation of lipid droplets in cerebral cortex and SVZ. These data indicate that exposure to EE and estrogen treatment can induce lipolysis and reduce abnormal lipid accumulation in the liver and various brain regions.

### EE and Estrogen Treatment Can Increase the Level of βHB in OVX Mice

**Figure 9:**
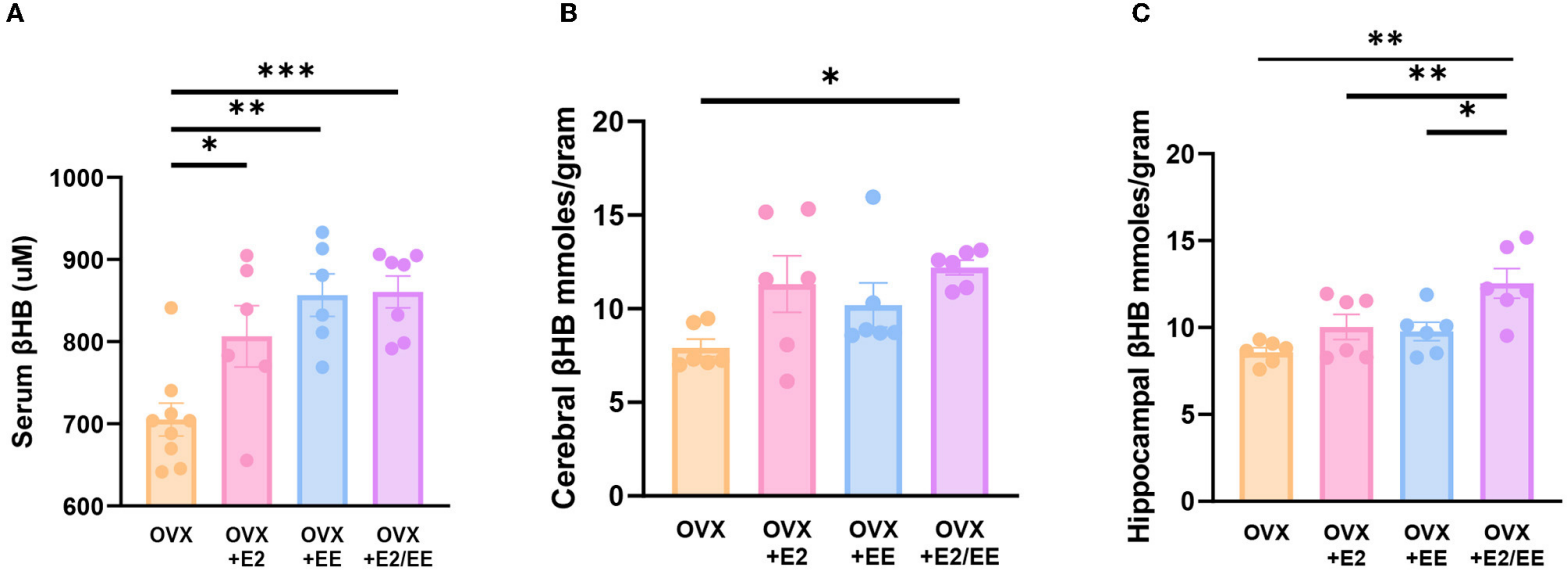
Exposure to EE and estrogen treatment can increase the level of βHB in OVX mice. **(A)** Serum βHB (*n* = 6–9 per group). **(B)** Cerebral cortex βHB (*n* = 6 per group). **(C)** Hippocampus βHB (*n* = 6 per group). One-way ANOVA with Tukey multiple comparison test. Data represented are means ± SEM. *p < 0.05, **p < 0.01, and ***p < 0.001.

βHB ELISA analysis indicated a significant increase in serum βHB after estrogen treatment and exposure to EE (Figure 9A). The synergistic effect of estrogen and EE was observed on the βHB level in the cerebral cortex and hippocampus (Figures 9B,C). In response to the increased rate of lipolysis, the level of βHB was significantly increased by EE and estrogen treatment in serum and the brain regions. Particularly, in female mice with EE and estrogen treatment, the level of βHB in cerebral cortex was significantly increased compared with OVX mice (*P* = 0.0319, Figure 9B), and the level of hippocampal βHB was significantly increased compared with OVX mice (*P* = 0.0014, Figure 9C).

### EE and Estrogen Treatment Exerts Synergistic Effects on the Expression of βHB-Related Genes and Proteins in OVX Mice

**Figure 10:**
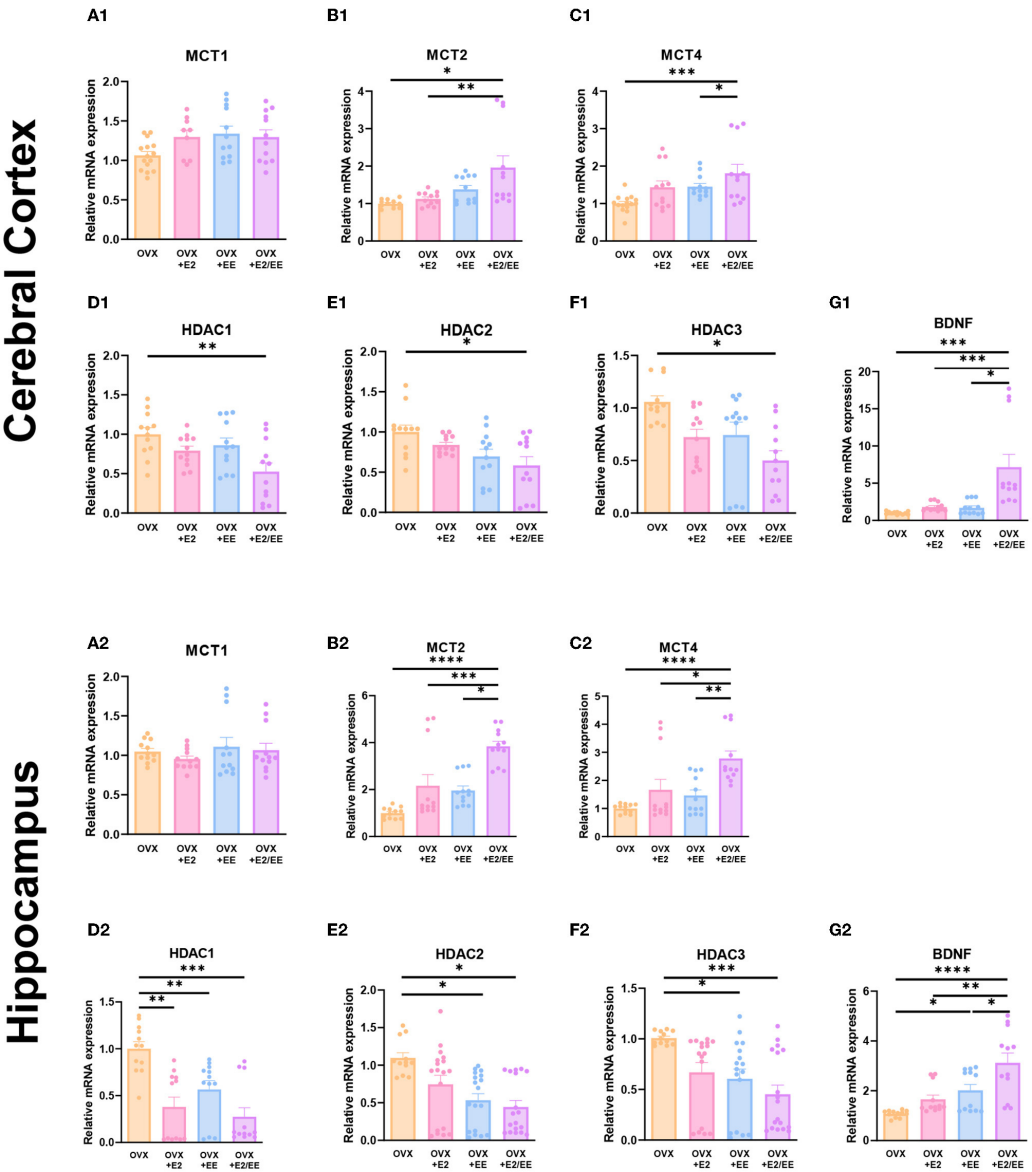
Synergistic effects of estrogen and EE on the expression of ketone-related genes by qRT-PCR. **(A1–G1)** Cerebral cortex and **(A2–G2)** hippocampus mRNA levels for βHB-related genes measured using qRT-PCR (*n* = 4 per group). All samples were run in triplicate. One-way ANOVA with Tukey multiple comparison test. Data represented are means ± SEM. *p < 0.05, **p < 0.01, ***p < 0.001, and ****p < 0.0001.

**Figure 11:**
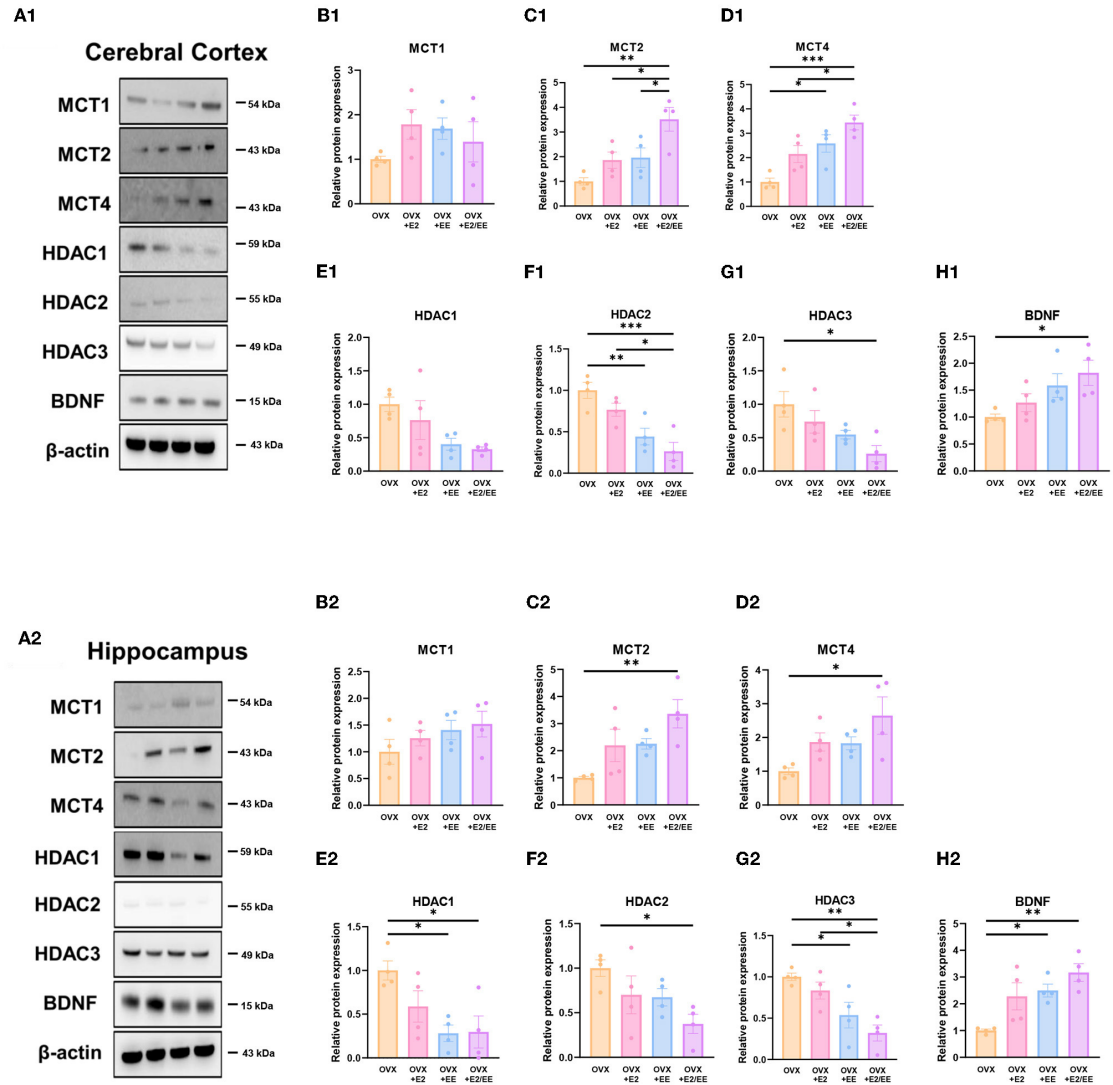
Synergistic effects of estrogen and EE on the expression of ketone-related proteins by western blot. **(A1–H1)** Cerebral cortex and **(A2–H2)** hippocampus protein levels for βHB-related proteins measured using western blot (*n* = 4 per group). One-way ANOVA with Tukey multiple comparison test. Data represented are means ± SEM. *p < 0.05, **p < 0.01, and ***p < 0.001.

To examine the expression of βHB-related genes, qRT-PCR was conducted in the cerebral cortex (Figures 10A1–G1) and hippocampus (Figures 10A2–G2). The significantly synergistic effect of EE and estrogen treatment was noted in MCT2, MCT4, HDAC1, HDAC2, HDAC3, and BDNF in both cerebral cortex and hippocampus. To examine the expression of βHB-related proteins, western blotting was conducted. The representative western blot images of βHB-related proteins in cerebral cortex and hippocampus are shown in Figures 11A1,A2, respectively. The significantly synergistic effect of EE and estrogen treatment was noted in MCT2, MCT4, HDAC2, HDAC3, and BDNF in cerebral cortex (Figures 11B1–H1). Moreover, the significantly synergistic effect of EE and estrogen treatment was noted in MCT2, MCT4, HDAC1, HDAC2, HDAC3, and BDNF in hippocampus (Figures 11B2–H2). Collectively, these data indicate that the expression of βHB-related genes and proteins was significantly regulated by exposure to EE and estrogen treatment. Particularly, the expressions of BDNF genes and proteins were significantly enhanced in the cerebral cortex (*P* = 0.0004, Figure 10G1; *P* = 0.0342, Figure 11H1) and hippocampus (*P* < 0.0001, Figure 10G2; *P* = 0.0025, Figure 11H2) of the female mice.

### EE and Estrogen Treatment Exerts Synergistic Effects on βHB Uptake of the Brain by Both Astrocytes and Neurons in OVX Mice

**Figure 12:**
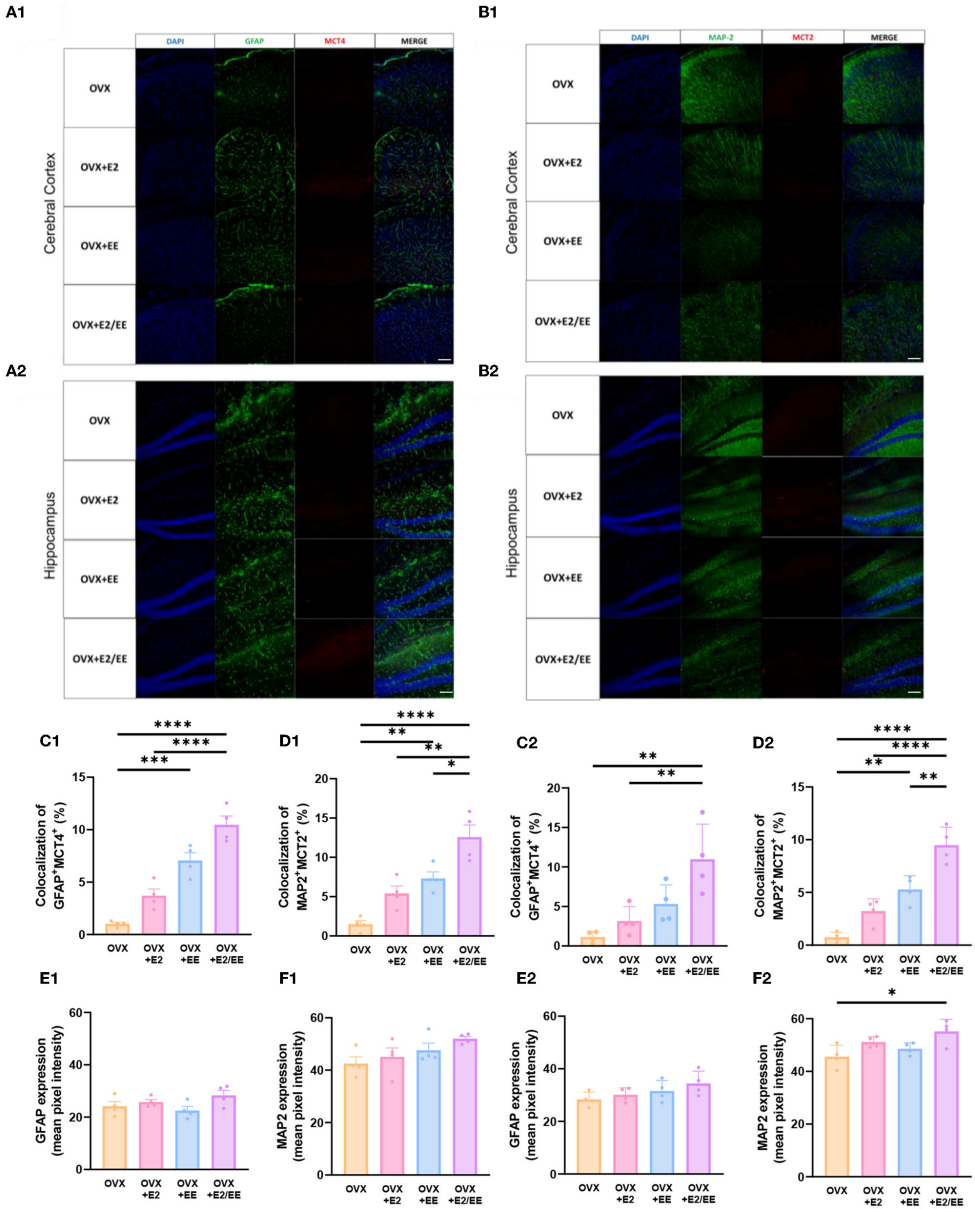
Synergistic effects of estrogen and EE on the uptake of βHB in cerebral cortex and hippocampus. Representative IHC images of **(A1,A2)** GFAP^+^MCT4^+^ in cerebral cortex and hippocampus and **(B1,B2)** MAP2^+^MCT2^+^ in the cerebral cortex and hippocampus (*n* = 4 per group). **(C1–F1)** Quantification of GFAP^+^MCT4^+^ and MAP2^+^MCT2^+^ in the cerebral cortex and hippocampus. **(C2–F2)** Raw intensity of GFAP and MAP2 in cerebral cortex and hippocampus. One-way ANOVA with Tukey multiple comparison test. Data represented are means ± SEM. *p < 0.05, **p < 0.01, ***p < 0.001, and ****p < 0.0001. A white bar = 50 μm.

To examine the uptake of βHB in cerebral cortex and hippocampus, histological assessments with GFAP, MCT4, MAP-2, and MCT2 were conducted. The representative images of GFAP and MCT4 in the cerebral cortex and hippocampus are shown in Figures 12A1,A2, respectively. The representative images of MAP-2 and MCT2 in the cerebral cortex and hippocampus are shown in Figures 12B1,B2, respectively. The colocalization percent of GFAP with MCT4 (*P* < 0.0001, Figure 12C1) and MAP2 with MCT2 (*P* < 0.0001, Figure 12D1) in the cerebral cortex was significantly increased in OVX+E2/EE group compared to OVX group. In similar fashion, the colocalization percent of GFAP with MCT4 (*P* = 0.0012, Figure 12C2) and MAP2 with MCT2 (*P* < 0.0001, Figure 12D2) in the hippocampus was significantly increased in OVX + E2/EE group compared to OVX group (Figures 12C2,D2). Raw intensity of GFAP and MAP2 for cerebral cortex and hippocampus are shown in Figures 12E1,F1,E2,F2. These combined data indicate that long-term exposure to EE and estrogen treatment can synergistically induce the higher βHB uptake in cerebral cortex and hippocampus by astrocytes and neurons in OVX mice.

### EE and Estrogen Treatment Synergistically Improve Motor, Emotional, and Cognitive Functions in OVX Mice

**Figure 13:**
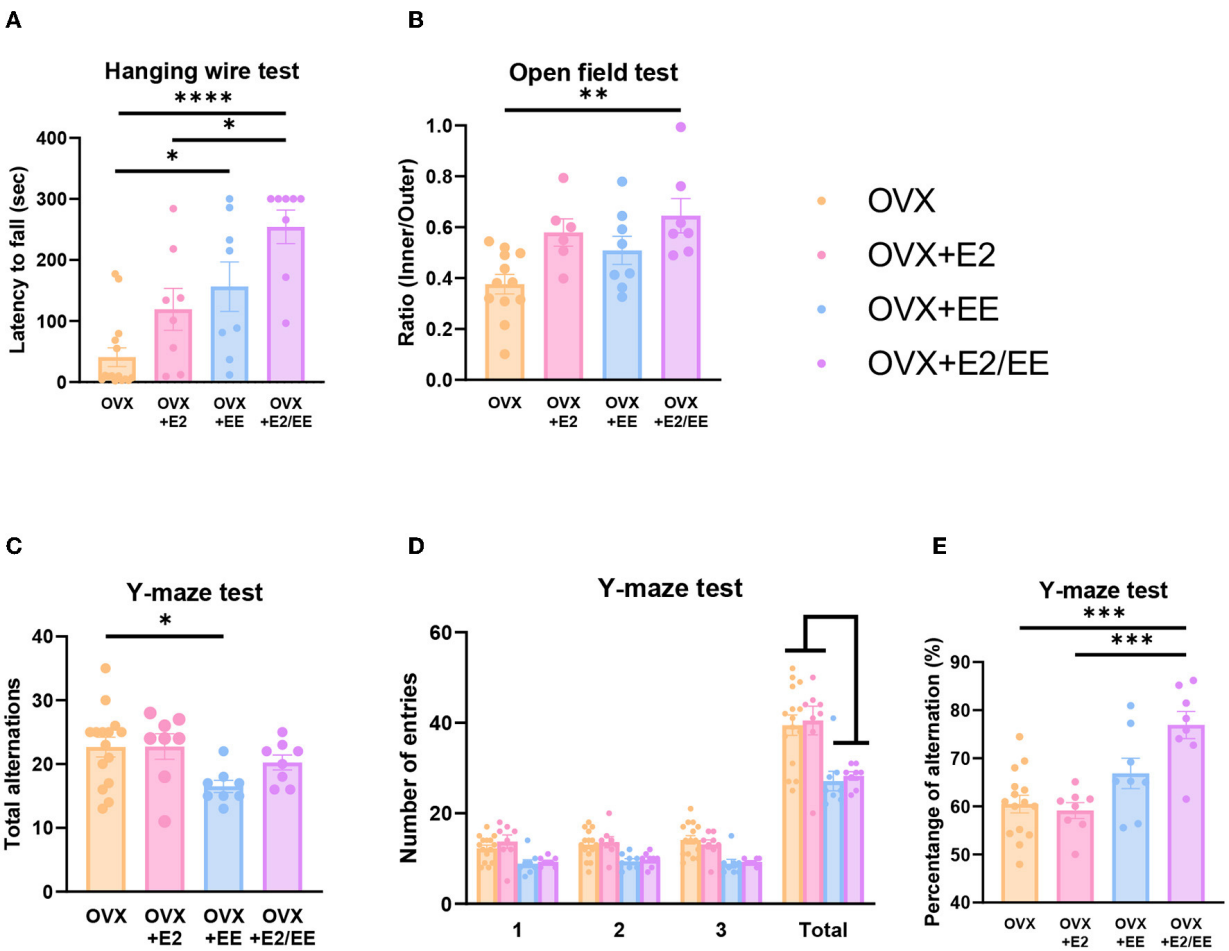
Synergistic effects of estrogen and EE on functional improvement in OVX models. Behavioral test assessments conducted in the OVX groups. **(A)** Hanging wire test, hippocampus (*n* = 8–15 per group). **(B)** Open field test (*n* = 6–12 per group). **(C)** Number of alternative behaviors, **(D)** number of entries, and **(E)** percent alternation of Y-maze test (8–15 per group). One-way ANOVA with Tukey multiple comparison test. Data represented are means ± SEM. *p < 0.05, **p < 0.01, ***p < 0.001, and ****p < 0.0001.

To examine motor, emotional, and cognitive function of OVX mice, hanging wire test, open-field test, and Y-maze test were conducted. Significant improvement in motor function following estrogen treatment and exposure to EE was observed in the hanging wire test (*P* < 0.0001, Figure 13A). Moreover, a significant reduction in anxiety level (*P* = 0.0036, Figure 13B) and cognitive improvement (*P* = 0.0001, Figure 13E) were observed in OVX mice after treatment with estrogen and EE. Raw number of alternative behaviors and number of entries in Y-maze are shown in Figures 13C,D, respectively. Using an OVX model, these data indicate that EE and estrogen treatment synergistically regulate the expression of βHB-related genes and proteins, thereby improving motor, cognitive, and emotional functions in female mice.

## Discussion

EE intervention is a non-invasive strategy for improving metabolic and brain function (Briones et al., 2013; Seo et al., 2018; De Souza et al., 2019; Queen et al., 2020). However, despite its therapeutic potential, many previous studies have reported that a large amount of variation exists in the effects of EE by sex, sex-related differences are observed in metabolic pathways, especially lipid metabolism, under nutrient stress (Mittendorfer et al., 2001; Soeters et al., 2007). Since females generally have more body fat than males do, females have a propensity for the increased oxidation of adiposity, and have more enzymes responsible for β-oxidation (Blaak, 2001; Maher et al., 2010). Since estrogen and exposure to EE are two of the most significant lipolysis factors that influence body composition and metabolism, it is important to consider the interrelated interactions of these factors.

In this study, we investigated metabolic responses after the long-term exposure to EE based on sex. DXA and blood biochemical analyses of fat content indicated that fat reduction occurred after the long-term exposure to EE regardless of sex. Analyses of indirect calorimetry indicated that exposure to EE and estrogen levels can change body composition, consistent with previous studies demonstrating young females have overall higher core temperature than males, which is also influenced by estrogen levels (Heled et al., 2001; Kaciuba-Uscilko and Grucza, 2001; Sanchez-Alavez et al., 2011). Previous results also showed that long-term exposure to EE or prolonged exercise can reduce the fat content in the whole body (Cao et al., 2011; Swift et al., 2014). Although the exact mechanism of EE-induced fat reduction is not clear, enhanced fat oxidation and improved glucose tolerance are responsible for the underlying mechanism (Goodpaster et al., 2003; Solomon et al., 2008). In the estrogen-deficient model, abnormal fat accumulation was observed in liver and brain regions (Supplementary Figures 2A,B). This accumulation was significantly alleviated by estrogen and EE exposure.

In response to the fat reduction in both models, the level of βHB was significantly upregulated in both the serum and brain regions by estrogen and EE exposure in this study. Moreover, the expression of βHB-related genes and proteins was significantly modulated by estrogen levels and exposure to EE. This alteration may induce behavioral improvements in motor, cognitive, and emotional functions.

It is important to note that young females utilize larger amounts of fatty acids to produce ketone bodies than their male counterparts under nutritional stress (Marinou et al., 2011; Ballestri et al., 2017). Moreover, in rodent model studies, there were sex-specific and strain-specific effects of calorie restriction on circulating βHB (Mitchell et al., 2016). Our result indicated that circulating βHB concentrations were higher in females than in males, regardless of the housing conditions. Interestingly, further subgroup analysis based on estrus cycle in the female group indicated that significantly higher circulating βHB levels following EE exposure at the D/M stage was observed compared to that in female control mice at the D/M stage. Previous studies have shown that estrogen levels are closely associated with mitochondrial β-oxidation of fatty acids (Oliveira et al., 2018), and estrogen regulates the level of histone acetylation associated with memory consolidation and increases BDNF promoter acetylation (Fortress et al., 2014). These combined results may suggest the synergistic effect of estrogen and exposure to EE on the utilization of βHB and BDNF, contributing to the effects of EE on metabolism and brain function under the influence of estrogen.

MCTs are solute carrier transporters of alternative metabolites, such as lactate, pyruvate, and ketone bodies (Vijay and Morris, 2014). MCTs are responsible for brain energy metabolism, and three MCT isoforms have been identified in the brain: MCT1, MCT2, and MCT4. MCT1, MCT2 and MCT4 show prominent expression in brain endothelial cells, neurons, and astrocytes, respectively (Pierre and Pellerin, 2005). The expression of these transporters is detectable in varying amounts in the cerebral cortex and hippocampus (Halestrap, 2012; Halestrap and Wilson, 2012). In this study, the higher colocalization of MCT2^+^MAP2^+^ and MCT4^+^GFAP^+^ was observed with exposure to EE and estrogen, and the higher magnification of these images is presented in Supplementary Figure 2B. Previous studies have shown a close interrelationship among the levels of βHB, MCTs, HDACs, and BDNF (Robinet and Pellerin, 2011; Halestrap and Wilson, 2012; Takimoto and Hamada, 2014; Sleiman et al., 2016; Achanta and Rae, 2017; Puchalska and Crawford, 2017; Li et al., 2020). Our qRT-PCR and western blotting analyses indicated the synergistic effects of estrogen and EE on the expression of MCT2 and MCT4. Similarly, the expression of HDAC1, 2, and 3 was significantly lower, and the expression of BDNF was significantly higher. The physiological significance of MCT2 and MCT4 on brain energy metabolism and neuroplasticity has been noted in the murine model (Pierre et al., 2002; Pellerin et al., 2005). The inhibition of MCT2 can impair long-term memory, and MCT4 knockout can kill various cancer cells (Newman et al., 2011; Benjamin et al., 2018; Fang et al., 2022).

An estrogen deficient model can be created using ovariectomy (Yokose et al., 1996). Estrogen-deficiency can induce spatial memory impairment, anxious, and depressive behaviors (Lagunas et al., 2010; Djiogue et al., 2018). Short-term estrogen treatment can address these physical and psychological stress-induced cognitive impairments (Khayum et al., 2020; Khaleghi et al., 2021), and prolonged regular (involuntary) exercise can improve estrogen levels in various brain regions and motor coordination performance (Rauf et al., 2015). Exercise also exerts the similar effects of estrogen in terms of lipid oxidation, fat reduction, and inflammation regulation in OVX mice (Jackson et al., 2011; Pighon et al., 2011; Gorres-Martens et al., 2018; Fuller et al., 2021). Moreover, functional locomotor improvement following estrogen treatment was observed in OVX mice. Cognitive deficits induced by decreased BDNF levels can be alleviated by voluntary exercise and estrogen therapy, which regulate the expression of histone deacetylases (Pedram et al., 2013; Rashidy-Pour et al., 2019). These data indicate that estrogen and exercise can improve behavioral functions by enhancing the metabolic phenotype. Using an OVX model in this study, the effect of EE and estrogen treatment proved the hypothesis that EE upregulates βHB and BDNF underlying functional improvement in female mice.

This study has several limitations that some inconsistencies exist in the normal model due to a small size sample, and complexity in defining an absolute EE, which contains complex inanimate and social stimulations. Many components within a complex definition of EE make it difficult to discern which stimulation contributes most to the improvements. Since this study only focused on the effect of EE and estrogen on metabolism, further studies with orchidectomized mice should be conducted to see the holistic sex-specific effect.

In conclusion, this EE exerts an effect on both males and females. However, females have shown significantly differential results in MCT2, HDAC2, and BDNF in cerebral cortex and MCT2, MCT4, HDAC1, HDAC2, BDNF in hippocampus. The level of MCTs, HDACs, and BDNF in the cerebral cortex and hippocampus were regulated by exposure to EE and influenced by the estrogen level. In response to the changed level of βHB, the behavioral improvements in motor and cognition were observed in normal and OVX mice. These combined events showed that EE induces metabolic, molecular, and behavioral changes under the influence of sex, partially by the estrogen level. These findings may be applied to the sex-specific modification of EE and neuroplasticity in female mice from the EE treatment.

## Data Availability Statement

The original contributions presented in the study are included in the article/Supplementary Material, further inquiries can be directed to the corresponding author/s.

## Ethics Statement

The animal study was reviewed and approved by the Association for Assessment and Accreditation of Laboratory Animal Care and the Institutional Animal Care and Use Committee of the Yonsei University Health System (permit number: 2018-0110, 2019- 0336, and 2020-0054). All procedures were in accordance with the guidelines of the National Institutes of Health's Guide for the Care and Use of Laboratory Animals. These regulations, notifications, and guidelines originated and were modified from the Animal Protection Law (2008), Laboratory Animal Act (2008), and Eighth Edition of the Guide for the Care and Use of Laboratory Animals (NRC 2011).

## Author Contributions

SP acquired funding for the work, designed the study, developed the setup, drafted the manuscript, and performed the experiments. JK and JH performed experiments and confirmed the accuracy of the data. JHH performed DXA scan and analysis. KK contributed to data validation, data curation, and graphic illustration. S-RC acquired funding for the work, interpreted the data, wrote the manuscript, and conducted study supervision. All authors contributed to the article and approved the submitted version.

## Funding

This research was supported by the Korean Fund for Regenerative Medicine (KFRM) grant funded by the Korea government (the Ministry of Science and ICT, the Ministry of Health & Welfare). (21A0202L1 and 21C0715L1) to S-RC, a grant from the Korean Health Technology R&D Project through the Korea Health Industry Development Institute (KHIDI), funded by the Ministry of Health & Welfare, Republic of Korea (HI21C1314) to S-RC. SP received funding from Hyundai Motor Chung Mong-Koo Foundation. The funder was not involved in the study design, collection, analysis, interpretation of data, the writing of this article or the decision to submit it for publication.

## Conflict of Interest

The authors declare that the research was conducted in the absence of any commercial or financial relationships that could be construed as a potential conflict of interest. The reviewer SK declared a shared affiliation with the authors to the handling editor at the time of review.

## Publisher's Note

All claims expressed in this article are solely those of the authors and do not necessarily represent those of their affiliated organizations, or those of the publisher, the editors and the reviewers. Any product that may be evaluated in this article, or claim that may be made by its manufacturer, is not guaranteed or endorsed by the publisher.

## Supplementary Material

Supplementary Figure 1 Experimental methods for the study. **(A)** The real picture of an EE cage and a control cage. **(B)** Determination of estrus cycle by vaginal cytology [(A) proestrus, (B) estrus, (C) diestrus, (D) metestrus]. **(C)** Ovariectomy [(A,B) ovary with fat tissues, (C) unilateral ovariectomy, (D) bilateral ovariectomy].

Supplementary Figure 2 Higher magnified images of **(A)** MAP-2^+^MCT2^+^ and **(B)** GFAP^+^MCT4^+^ in the OVX groups.

Supplementary Table 1 Primer sequences for qRT-PCR.
