# Supplementary material for: Environmental Enrichment and Estrogen Upregulate Beta-Hydroxybutyrate Underlying Functional Improvement
Source: Front Mol Neurosci. 2022 May 3;15:869799. doi: 10.3389/fnmol.2022.869799 (PMC9113201; doi:10.3389/fnmol.2022.869799)
Supplement: Supplementary Table 1 — Primer sequences for qRT-PCR. [file Table_1.DOCX]

**Supplementary table 1**: Primer sequences for qRT-PCR

| **Gene** | **Forward primer** | **Reverse primer** |
| --- | --- | --- |
| *MCT1* | TTGGGTTCTGTGTCTACGCC | GGCAGCATTCCACAATGGTC |
| *MCT2* | GTCGTAGTCTGTGCGTCCTT | CCAAGCGATCTGACTGGAGG |
| *MCT4* | CTCTTTGTGCCTCCGGTCTT | AGGTAGACAGAGTAGGGCCG |
| *BDH1* | TGTCAAGGTCAGTGTGGTGG | ACCGCTGTTGCAGTAGGTTT |
| *HDAC1* | ACCATCAAAGGACACGCCAA | ACCGCTGTTTCGTAAGTCCA |
| *HDAC2* | CGGCAAGAAGAAAGTGTGCTAC | TTTCTTCAGCAGTGGCTTTATGAG |
| *HDAC3* | TGCCCCAGATTTCACACTCC | GACACTGGGTGCATGGTTCA |
| *BDNF* | GGGTCACAGCGGCAGATAAA | ATTGCGAGTTCCAGTGC |
| *GAPDH* | CATCACTGCCACCCAGAAGACTG | ATGCCAGTGAGCTTCCCGTTCAG |

MCT, Monocarboxylate transporter; BDH, Beta-hydroxybutyrate dehydrogenase; HDAC, Histone deacetylase; BDNF, Brain-derived neurotrophic factor; GAPDH, Glyceraldehyde 3-phosphate dehydrogenase used as an internal control.
